# Relationship between clinician-level attributes and implementation outcomes from the Pathways to Comorbidity Care training program

**DOI:** 10.1186/s12909-022-03671-6

**Published:** 2022-08-05

**Authors:** Eva Louie, Vicki Giannopoulos, Andrew Baillie, Gabriela Uribe, Katie Wood, Maree Teesson, Paul S. Haber, Kirsten C. Morley

**Affiliations:** 1 Sydney Medical School, Faculty of Medicine and Health, The University of Sydney, Camperdown, NSW Australia; 2 Edith Collins Centre (Translational Centre in Alcohol, Drugs & Toxicology), Sydney Local Health District & Royal Prince Alfred Hospital, Camperdown, NSW Australia; 3 School of Health Sciences, Faculty of Medicine and Health, The University of Sydney, Camperdown, NSW Australia; 4 Menzies Centre for Health Policy, Faculty of Medicine and Health, The University of Sydney, Camperdown, NSW Australia; 5 The Matilda Centre for Research in Mental Health and Substance Use, The University of Sydney, Camperdown, NSW Australia; 6 Addiction Medicine, Central Clinical School, Sydney Medical School, University of Sydney, Camperdown, NSW 2006 Australia

**Keywords:** Implementation, Alcohol, Substance use, Clinician characteristics, Australia

## Abstract

**Background:**

The process of determining the best strategy for increasing the uptake of evidence-based practice might be improved through an understanding of relevant clinician-level factors. The Pathways to Comorbidity Care (PCC) training program (Louie E, et al., J Dual Diagnosis 17:304–12, 2021) aimed to facilitate integrated management of comorbid drug and alcohol and mental disorders amongst drug and alcohol clinicians. We hypothesised that uptake of integrated management of comorbidity following the implementation of the PCC program would be associated with clinician-level: (i) demographics (gender, education, experience), (ii) attitudes (evidence-based practice, therapist manuals, counselling self-efficacy), and (iii) organisational readiness to change.

**Methods:**

Twenty clinicians participated in the 9-month PCC training program. Attitudes towards evidence-based practices and psychotherapist manuals, self-efficacy, and organisational readiness to change, along with demographics, were measured at baseline. At follow-up, change in *Comorbidity Practice (CoP)* scores related to integrated comorbidity management were obtained using a file audit checklist and categorised into *high* (at least 60% increase in CoP), *medium* or *low* (a decrease of − 20% or less in CoP). Clinician-level characteristics were examined across the implementation categories.

**Results:**

There were no significant differences found between implementation groups on sociodemographic variables (p’s > 0.30), attitudes to evidence-based practices, attitudes to therapist manuals, and self-efficacy (p’s > 0.52). The high implementation group demonstrated significantly higher scores on *leadership practices* aspect of organisational readiness to change relative to the low and medium implementation group ((F(2, 16) = 3.63, *p* = 0.05; Cohen’s d = .31) but not on the other subscales (p’s > 0.07).

**Conclusions:**

Confidence that leadership will play a positive role in the implementation process may improve effectiveness of comorbidity training programs for drug and alcohol clinicians. On the other hand, contrary to our hypothesis, counselling self-efficacy, evidence-based practice attitudes, attitudes towards therapist manuals, gender, education and experience were not distinguishing factors.

## Background

There is a growing consensus in the literature regarding the role of evidence-based practice (EBP) implementation in improving public healthcare provision [1–6]. These implementation processes are particularly challenging within the human services, where technological innovations are delivered by individuals and organisations operating within complex and multi-level systems of influence [3, 7]. The drug and alcohol treatment setting exemplifies this complexity [8]. While there are many factors that may mediate the process of implementing EBPs in drug and alcohol treatment settings, it is important to consider that individual clinicians have a considerable amount of autonomy regarding the decision to adopt and implement a new EBP [9]. Clinicians asked to adopt EBPs related to comorbid drug and alcohol and mental health disorders, for instance, have been hesitant to treat mental health issues, which may be due to a history of siloed drug and alcohol and mental health services and a lack of mental health training [10]. Research into the effects of provider-level characteristics on implementation outcomes within substance use disorder settings is limited [11]. One approach has been to examine the extent to which these factors influence treatment delivery fidelity of evidence-based interventions following implementation efforts.

From a theoretical perspective, social cognitive theories have been widely used to explain health-related behaviours of individuals [12]. Although very little research had previously applied social cognitive theories to the study of health practitioner behaviour [13], these theories have been more frequently incorporated into implementation research over the past decade (e.g. [14–16]), and research evaluating EBP implementation in drug and alcohol settings has often been guided by the assumption that clinician factors have an important relationship to implementation fidelity [17]. Clinician factors most frequently measured in such studies include demographics (e.g. gender, age, experience, education, [18–33]), knowledge [25, 31, 33] and attitudes [18, 21, 22, 26, 27, 29]). Organisational change theories such as Rogers’ [34] diffusion of innovations theory have also highlighted the importance of clinician perceptions of the capacity of their organisation to support and implement new innovations, and drug and alcohol clinician perceptions of factors related to their organisational context have been evaluated as a possible mediator of implementation fidelity [18].

While clinician demographics have frequently been included in studies investigating factors related to implementation fidelity in drug and alcohol contexts, significant relationships are not often found [17]. Outcomes of studies investigating the relationship between drug and alcohol clinician knowledge and attitudes and implementation fidelity have been mixed. For instance, some studies have demonstrated that clinicians who are prepared for change and who have positive attitudes to EBP are more likely to implement such practices [18, 35–37], while others have not found a strong relationship between treatment fidelity and attitudes such as interest, confidence and commitment to EBPs [21, 29, 38]. Clinician perceptions of the organisational climate comprise a distinct set of beliefs found to influence implementation outcomes in drug and alcohol settings [29, 39].

The Pathways to Comorbidity Care (PCC) project evaluated the implementation of a multi-modal training package designed to improve *Clinician Practice* (identification and treatment), confidence (self-efficacy), knowledge and attitudes to comorbid substance use and mental disorders [40]. The PCC program was designed to upskill clinicians in an evidence-based, integrated management approach to the treatment of comorbidity. An integrated approach suggests that both the substance use and the mental health disorders are treated by the same clinician or within the same service. The PCC training included both didactic (seminar day and website with comorbidity resources) and interactive (clinical champion run group workshops and individual supervision) components. We have previously reported that the training package improved the percentage of clinical files demonstrating identification and management of comorbidity, self-efficacy, and attitudes toward screening and monitoring of comorbidity [41]. Barriers and facilitators of the PCC program have also been reported previously [42]. Specific facilitators of the implementation included characteristics of the intervention (credible source, uncomplicated approach, convincing evidence and quality design), a good consideration of patient needs, factors within the organisation (positive learning environment, leadership engagement), and the use of clinical champions. Mixed results were found with regards to clinician characteristics whereby self-efficacy was a strong facilitator, while specific personal beliefs and attitudes were implementation barriers [42].

Given inconsistencies in the existing literature regarding the role of clinician demographics and attitudes in facilitating implementation, in this study we aimed to examine the relationship between clinician characteristics on EBP implementation in the PCC program through a secondary analysis of data from the main study. We hypothesised that high implementation would be associated with characteristics across two domains: (i) attitudes (positive attitudes to evidence-based practice and therapist manuals, higher counselling self-efficacy), and (ii) more positive perceptions of organisational readiness to change. We also included demographics (higher levels of education, increased experience) in our analysis to explore any possible relationships present in this sample.

## Methods

Details of this multi-modal training package have been published previously [43]. Findings reported here are based on a secondary analysis of data obtained in the original study.

### Recruitment

The study was approved by the Human Ethics Review Committees of the Sydney Local Health District, South Western Sydney Local Health District, Central Coast Local Health District, Hunter New England Research Ethics and Governance Office which covered two participating services, and Mid North Coast Local Health District (X16-0440 & HREC/16/RPAH/624).

Six outpatient and community drug and alcohol services within the NSW Health system in New South Wales, Australia, participated in the Pathways to Comorbidity Care (PCC) project. Three of the six services participated in the PCC training program and the remaining three formed the control group for the study. Findings reported here only pertain to the PCC training group.

In total, 29 eligible clinicians from the PCC training group returned consent forms. Of the 29 participants initially enrolled in the study, 20 completed the study. Clinicians in the PCC condition completed baseline and follow-up assessments, including an interview related to their participation in the training. All sites provided clinical notes at baseline and follow-up, which were used to assess practice change. Only the baseline assessment and practice change assessment are examined in this study.

### Measures

A comprehensive account of assessments has been provided previously [44]. Measures relevant to this study include: the adapted version of the Personnel Data Inventory (an index of demographic and professional information; [39]); the Evidence-Based Practice Attitudes Scale (EBPAS; [45]) which evaluates four aspects of attitude towards evidence based practice (i.e. intuitive appeal, likelihood of adopting if required to, openness to new practices and perceived divergence of practice with evidence-based practice), and has been found to have a moderate to excellent reliability [46]; the Survey of Attitudes to Therapist Manuals (SATM) which addresses experience with treatment manuals, attitudes towards treatment manuals, and beliefs about the content of treatment manuals [47], and has been validated psychometrically using factor analysis and internal consistency [47]; the Addiction Counseling Self-Efficacy Scale (ACSES: [48]) which assesses self-efficacy of treating comorbid drug and alcohol and mental health problems, and factor analyses have supported the validity of the subscales and total score [48]; and the Organizational Readiness for Change Assessment Tool (ORCA: [49]) which measures organisational readiness for implementing practice change in healthcare settings, and is considered a valid and reliable measure [50]. File audits of clinical notes made during the 3 months prior to baseline and follow-up (10 files per clinician) were evaluated using a checklist for Comorbidity Practice (CoP). This checklist assessed four relevant practice themes for presence and quality: screening, assessment, treatment and referral.

### Analysis

Participants were divided into *low, medium* and *high* implementers based on the degree of change in CoP between baseline and follow-up. CoP scores were derived from file audits of clinical notes (10 per clinician at each time point), which were evaluated using a checklist of important practice themes (screening and monitoring, assessment, treatment and referral) by one researcher and one clinician (clinical supervisor). Previously studies of the PCC program have evaluated Clinical Practice by determining the percentage of total clinical files demonstrating identification and treatment of comorbidity over a set rate. For the current study, we assessed clinical practice change in greater depth by deriving a *Comorbidity Practice (CoP)* score from the presence and quality of a checklist of integrated comorbidity management themes in clinical files. For each theme, scores ranged from “0” not present, to “1” evidence of practice theme but lacking subsequent details, to “2” a detailed account of the practice theme. Scores for each clinician and set of files were then discussed and an agreement reached in cases where the scores did not align. A total CoP score was calculated by creating a composite of the four themes.

Low, medium and high categories were derived using visual binning, and it was determined that a conservative significance level of *p* < .01 would be required between groups to ensure that they were distinct. Binning is a statistical process of converting a continuous variable into a discrete form. In this instance, variables were binned according to perentiles. Following binning, 5 categories were identified. The highest only included one participant and was therefore grouped with category 4. Category 2 and 3 did not differ significantly enough and were also combined. The three categories created through this process (*low, medium* and *high*) were distinctly different. The low group included participants with CoP scores that had changed by − 20% or less following training, the high group included participants with CoP scores that had changed by 60% or more following training, and the medium group included participants between these extremes. These categories also made sense clinically, since there was a clear distinction between being less likely to practice integrated management techniques, remaining unchanged, and incorporating many more integrated management practices.

Continuous and categorical variables were examined using ANOVAS and Chi square tests respectively to examine differences between high, medium and low implementation groups for baseline CoP scores and across the three domains: demographics (age, sex, education, and professional role); attitudes (evidence-based practice attitudes (EBPAS), attitudes towards therapist manuals (SATM), addiction counselling self-efficacy (ACSES)); and perceptions of organisational readiness to change (organisational readiness to change (ORCA)). Between group differences on these scales were evaluated using effect sizes (Cohen’s d) and 95% confidence intervals. Data were analysed using SPSS 28 for Mac OSX.

## Results

### Sample characteristics

Implementation change scores were derived from measures of CoP obtained from participants across all 3 PCC sites. The 20 clinicians that participated in the PCC training were grouped according to distinct patterns of change in CoP (*N* = 4 low implementation, *N =* 10 medium implementation, *N* = 6 high implementation). Baseline clinician-level characteristics are displayed in Table 1. The overall mean age was 51.53 (*SD* ± 8.14) years, 75% were female. Most participants had completed a university degree (60%). The most common professional role was psychologist (45%) and the mean number of years since graduating was 15.46 (*SD* ± 8.88).

**Table 1:** Baseline Characteristics of High, Medium and Low Implementers

Variable	High Implementers (***n*** = 6)	Medium Implementers (***n*** = 10)	Low Implementers (***n*** = 4)
Age (m, SD)	51.33 (8.98)	51.00 (8.62)	52.75 (8.22)
Gender (%)			
Male	33.3	11.1	50
Female	66.7	88.9	50
Geographic location (%)			
Metro	50	88	50
Regional	50	12	50
Years since graduating (m, SD)	19.33 (10.50)	15.00 (10.27)	15.00 (5.29)
Highest level of education (%)			
Bachelor’s Degree	83.3	55.6	50
Post-graduate Degree	16.7	44.4	50
Occupation (%)			
Psychologist	16.7	55.6	50
Social worker	33.3	–	25
Counsellor	–	22.2	25
Case worker	16.7	11.1	–
Nurse	16.7	11.1	–

Data represent mean + SD unless otherwise noted. There were no significant differences found between high, mediun and low implementers on baseline variables

### Comorbidity practice implementation scores

CoP scores are presented in Table 2. Across the entire sample, the mean CoP score at baseline was 1.02 (*SD* ± .83) and 1.25 (*SD* ± .81) at follow-up, with an overall mean increase of 23% from baseline. There was a 58% increase in assessment of comorbidity at follow-up along with a 35% increase for screening, 18% for treatment and 4% for referral. Differences in baseline CoP scores between the three implementation groups were not significant (*X*^*2*^ (2, *N* = 18) = 3.32, *p* = 0.06). The high implementation group demonstrated an increase in CoP of 75% following training, compared with 27% for the medium group and a decrease of 22% in the low group.

**Table 2:** Comorbidity Practice (CoP) mean scores for High, Medium and Low Implementers

Variable	High (***n*** = 6)	Medium (***n*** = 10)	Low (***n*** = 4)	Total (***n*** = 20)
CoP Baseline score				
Screening	.25 (0.22)	.09 (0.06)	.49 (0.42)	.23 (0.26)
Assessment	.11 (0.15)	.05 (0.06)	.31 (0.45)	.12 (0.23)
Treatment	.30 (0.27)	.41 (0.46)	.72 (0.51)	.44 (0.43)
Referral	.31 (0.24)	.13 (0.08)	.32 (0.53)	.23 (0.27)
Total score	.99 (0.76)	.67 (0.42)	1.83 (1.22)	1.02 (.83)
CoP Follow-up score				
Screening	.54 (0.35)	.11 (0.11)	.38 (0.34)	.31 (0.31)
Assessment	.27 (0.25)	.09 (0.12)	.29 (0.37)	.19 (0.23)
Treatment	.55 (0.38)	.46 (0.54)	.60 (0.77)	.52 (0.52)
Referral	.38 (0.25)	.19 (0.11)	.15 (0.17)	.24 (0.19)
Total score	1.73 (0.75)	.85 (0.45)	1.42 (1.18)	1.25 (.81)
CoP Change (%)				
Screening	116.00	2.00	−22.45	34.78
Assessment	145.46	80.00	−6.45	58.33
Treatment	83.33	12.20	−16.67	18.18
Referral	22.58	46.15	−53.13	4.35
Total score	74.75	26.87	−22.40	22.55

Data represent mean + SD. CoP scores indicate the degree of detail in CoP themes (screening, assessment, treatment and referral of comorbidity) found in clinical notes. Scores range from 0 to 2. Total CoP score represents the sum of each aspect. CoP Change represents the percentage of change in in CoP scores following training. *CoP* Comorbidity Practice. This checklist assessed four relevant practice themes for presence and quality: screening, assessment, treatment and referral

### Clinician-level factors and association with implementation scores

There were no significant differences between high, medium and low implementation groups regarding gender (*X*^*2*^ (2, *N* = 19) = 2.38, *p* = 0.30), education (*X*^*2*^ (4, *N* = 19) = 2.55, *p* = 0.57) or occupation (*X*^*2*^ (5, *N* = 19) = 8.14, *p* = 0.42) at baseline. Likewise, no differences were found between high, medium and low implementation groups in age or experience (*F*s < 0.42). Comparisons between high, medium and low implementation groups on clinician attitudes and perceptions at baseline are presented in Table 3. There were no significant differences between high, medium and low implementation groups on the EBPAS (F(2, 16) = .22, *p* = 0.81), the SATM positive scale (F(2, 16) = 0.01, *p* = 0.99) and negative scale (F(2, 16) = 0.08, *p* = 0.92) or the ACSES (F(2, 16) = 0.69, *p* = 0.52). Although there was also no significant difference between groups on the total ORCA (F(2, 16) = 2.37, *p* = 0.13), or on subscales including *leadership culture* (F(2, 16) = 3.21, *p* = 0.07)*, staff culture* (F(2, 16) = 0.61, *p* = 0.56)*, measurement* (F(2, 16) = 1.30, *p* = 0.30)*, opinion leaders* (F(2, 16) = 0.40, *p* = 0.68) *and resources* (F(2, 16) = .08, *p* = 0.92)*,* there was a significant difference found between groups on the *leadership behaviour* subscale of the ORCA (F(2, 16) = 3.63, *p* = 0.05; Cohen’s d = 0.31). A Tukey’ HSD test for multiple comparisons revealed that *leadership behaviour* was significantly different between high and low implementers (*p =* 0.05, 95% C.I. = − 14.03, 0.04). There was no significant difference between high and medium (*p* = 0.09), or medium and low implementation fidelity groups (*p* = 0.85).

**Table 3:** Effect Size Comparisons of Baseline Psychometric Variables between High, Medium and Low Clinical Practice Implementers

Baseline	High (***n*** = 6)	Medium (***n*** = 9)	Low (***n*** = 4)	Effect size (d), (95% CI)
	Mean (SD)	Mean (SD)	Mean (SD)	
EBPAS total	59.17 (10.05)	58.56 (6.89)	62 (11.02)	.03 (55.41–63.54)
PATM negative	29.67 (6.59)	29.78 (8.80)	30.50 (9.98)	.00 (22.74–36.59)
PATM positive	22.33 (4.23)	23.25 (3.92)	22.25 (7.32)	.01 (20.43–25.02)
ACSES total	117.33 (22.57)	120.56 (18.73)	131.00 (6.33)	.29 (112.98–141.02)
Organisational Readiness to change (context) Total	79.00 (14.93)	79.11 (10.86)	61.75 (19.48)	.23 (68.11–82.73)
Leadership culture	10.00 (6.75)	11.11 (2.26)	6.75 (4.57)	.29 (8.30–17.18)
Staff culture	15.67 (3.62)	15.56 (1.94)	14.00 (2.00)	.07 (14.04–16.49)
Leadership behaviour^*^	15.00 (3.03)	13.78 (3.70)	8.00 (6.58)	.31 (10.63–15.26)
Measurement	13.00 (3.58)	13.56 (2.24)	9.75 (7.14)	.14 (10.62–14.54)
Opinion leaders	14.17 (3.13)	14.22 (1.86)	13.00 (2.31)	.05 (12.83–15.07)
General resources	11.17 (2.79)	10.89 (4.20)	10.25 (2.63)	.01 (8.24–14.09)

Data represent mean + SD. * *p* < 0.05, significant difference between groups, ANOVA

*Abbreviations*: *EBPAS* Evidence-Based Practice Attitudes Scale (min 15 to maximum 75), *SATM* the Survey of Attitudes to Therapist Manuals (Negative scale minimum 10 to maximum 50, Positive scale minimum 7 to maximum 35), *ACSES* the Addiction Counseling Self-Efficacy Scale (minimum 32 to maximum 160), *ORCA* the Organizational Readiness for Change Assessment Tool (minimum 23 to maximum 115)

## Discussion

Understanding the interrelationships between clinician-level factors and the outcomes of implementation efforts may assist with the development of more effective implementation strategies. The aim of this study was to evaluate the effects of clinician-level characteristics on clinical practice outcomes of the PCC program implementation, which involved clinicians practicing counselling interventions in drug and alcohol outpatient clinics. Our hypotheses related to characteristics across the domains of attitudes and perceptions of organisational readiness to change, and we were interested to see whether clinician demographics were relevant to this sample. The only hypothesis confirmed by the results of this study pertained to the clinician’s perceptions of one aspect of organisational readiness to change.

With regards to clinician demographics, the results of this study were not able to provide any further clarity. Existing research suggests that years of experience [51] and higher levels of education are associated with higher fidelity [18]. However, this relationship between education and fidelity is sometimes no longer present following training [28], and clinicians with lower levels of education have been found to demonstrate greater increases in implementation fidelity following training [19]. It is therefore possible that participating in the PCC training attenuated any differences in clinicians’ comorbidity practice related to prior education.

Likewise, findings from this study did not provide any additional insight into the potential role of clinician attitudes in the uptake of EBPs. Previous research related to salient attitudes has found that low endorsement of disease belief models [18], higher self-efficacy [22, 52] and an increased belief in the efficacy of the intervention [22] have implications for implementation outcomes. Although the relationship between self-efficacy and comorbidity practice was not significant, there was a trend toward significance observed in this small population which would warrant further investigation in a larger sample. In terms of clinican attitudes toward EBP, our findings did not confirm those of previous studies, which have shown that clinicians who are prepared for change and who have positive attitudes to EBP [18, 35–37], and treatment manuals [51] are more likely to implement such practices. On the contrary, attitudes of clinicians in this study are more consistent with alternate evidence suggesting that attitudes such as interest, confidence, and commitment to EBPs do not have a strong relationship with treatment fidelity [21, 29, 38]. It is also possible that the EBPAS measure used in this study did not capture aspects of clinician attitudes specific to the drug and alcohol treatment setting, since recent developments in measuring attitudes toward EPBs have demonstrated some utility in enhancing the specificity of such measures (e.g. [53]).

Interestingly, there was a relationship between implementation and leadership behaviour. Amongst the various components of ORC that have been cited in the literature as influencing staff adaptation to new innovations (e.g. positive organisational climate [54], valuing innovation, creative and supportive leadership, and staff attributes [55]), findings from this study also suggest that perceptions of leadership are important. Specifically, clinicians who demonstrated the greatest changes in their comorbidity management practices strongly believed that senior leadership in their organisation effectively managed the continuous improvement of patient care, made the responsibilities of leadership and staff clear, actively promoted team cohesiveness and solved clinical care problems, and enhanced communication between relevant clinical services. This information is particularly helpful for developing implementation strategies, which may benefit from a better understanding of how individual clinicians perceive their leaders. These findings also emphasise the importance of engaging leadership when implementing a new EBP in drug and alcohol services, particularly with regards to the challenging area of comorbid drug and alcohol and mental health disorders.

Some practical suggestions for implementing strategies incorporating these findings might be to conduct a needs assessment prior to developing and implementing a training package, in which clinicians can provide confidential information about their perceptions of leadership. This information can inform leadership engagement and provide clues about possible challenges for particular leaders. The process of engaging leadership might also involve the development of particular strategies for promoting the new innovation such as identifying how it improves patient care, articulating how it will impact staff responsibilities, providing designated time for group workshops focused on supporting the delivery of the new innovation, and networking with other relevant clinical services.

### Strengths and Limitations

Firstly, the current findings have limited power due to the small sample size and there is a possibility that the effect size estimation is inflated. However, the broader sample was sourced from diverse geographic locations including a diverse representation of drug and alcohol outpatient clinicians across the Australian public health system. Secondly, the CoP outcome measure was based on the evaluation of clinician files rather than behavioural evaluations of comorbidity practice which may impact on the reliability of the measure. Nevertheless, the study does represent one of few attempts worldwide to evaluate the relationship between clinician-level factors and effectiveness of implementation of comorbidity training in the drug and alcohol field.

## Conclusion

These preliminary results suggest that drug and alcohol clinicians’ perceptions of their leaders has an influence on the extent to which they implement changes in their clinical practice following training and highlight the importance of engaging leadership in implementation efforts. In a broader sense, these findings challenge the notion that clinicians are largely responsible for barriers to implementation.

## Data Availability

The data that support the findings of this study are available from NSW Health (via Prof Paul Haber) but restrictions apply to the availability of these data, which were used under license for the current study, and so are not publicly available. Data are however available from the authors upon reasonable request and with permission of NSW Health.

## Abbreviations

EBP: Evidence-based Practice
ORC: Organisational readiness to change
PCC: Pathways to Comorbidity Care
NSW: New South Wales
EBPAS: Evidence-based Practice Attitudes Scale
SATM: Survey of Attitudes to Therapist Manuals
ORCA: Organizational Readiness to Change Scale
ACSES: Addiction Counseling Self-Efficacy Scale
CP: Comorbidity Practice
